# Prevalence of late antenatal care booking among pregnant women attending public health facilities of Kigamboni Municipality in Dar es Salaam region, Tanzania

**DOI:** 10.4314/ahs.v23i2.72

**Published:** 2023-06

**Authors:** Alana Ndomba, Moshi Ntabaye, Innocent Semali, Titus Kabalimu, Godwin Ndossi, Yohana Mashalla

**Affiliations:** 1 Department of Community Medicine, Hubert Kairuki Memorial University, P.O. Box 65300, Dar es Salaam, Tanzania; 2 Directorate of Postgraduate Studies & Research Institute, Hubert Kairuki Memorial University, Dar es Salaam, Tanzania

**Keywords:** Pregnant women, ANC, booking, parity, education

## Abstract

**Background:**

Good care during pregnancy is important for the health of mothers and development of the unborn baby. The study determined the prevalence and factors associated with late ANC booking among pregnant women at health facilities in Kigamboni Municipality in Dar es Salaam, Tanzania.

**Methods:**

This was an analytical cross-sectional study among pregnant women attending ANC services during second and third trimester in the selected health facilities. The study recruited 204 through convenient sampling. Multi-stage cluster sampling was used to select health facilities. A Standardised questionnaire was used to collect information through face-to-face interviews. Data was analysed using SPSS version 25.0. Proportions were used to estimate the magnitude of late ANC booking while bivariate and multivariate analyses were performed to determine factors associated with the magnitude of late ANC booking.

**Results:**

Late ANC bookings were high 174 (85.3%) among pregnant women who attended clinic week 13 and later compared to those who attended earlier than 13 weeks 30 (14.7%). Factors associated with likelihood for late ANC booking during the initial visit included tertiary education [AOR= 10.174, 95%CI: 1.002-103.301] and primigravida [AOR=0.101, 95%CI: 0.170-0.605].

**Conclusion:**

Majority of the pregnant women started ANC later than the recommended time. Health education provision at all community levels on the advantages and disadvantages of early and late ANC booking respectively should be strengthened.

## Introduction

The global maternal mortality has remained unacceptably high in many developing countries. In 2017, there were 295 000 reported women deaths during and following pregnancy and childbirth and majority of the deaths (94%) occurred in low and middle-income countries. Sub-Saharan Africa alone accounted for roughly two-thirds (196 000) of the maternal deaths 1. The risk of maternal mortality is highest for adolescent girls under 15 years old and complications of pregnancy and childbirth are higher among adolescent girls aged 10 – 19 years compared to women aged 20-24 2,3. Most of the causes of maternal deaths are detectable, preventable and treatable therefore, maternal mortality remains a priority agenda of the third UN Sustainable Development Goals 4.

Introduced in the 21st century, Antenatal Care (ANC) is among the key global strategies for reducing maternal and neonatal mortality and morbidity. ANC aims to detect and institute treatment of pregnancy related complications through appropriate medical and health education in order reduce the risks for maternal and neonatal mortality and morbidity 5,6. The World Health Organisation (WHO) has recommended a minimum of eight contacts: the first contact to take place in the first trimester (up to 12 weeks of gestation) 7. This recommendation aims to reduce maternal and perinatal deaths by increasing the opportunity of maternal and foetal assessment in order to identify complications and improve communication between the providers and the mothers 8–10. The packages provided during ANC visits include general physical and mental health examination, blood profile determination and screening for HIV and syphilis. Pregnant women found to be HIV infected become initiated on antiretroviral therapy (ART) at an early stage of pregnancy to prevent mother-to-child transmission (PMTCT) of HIV In addition, iron and vitamin supplements, and immunizations against tetanus if given early during pregnancy have shown to save lives of both mothers and infants^11^–^14^, reduce negative perinatal outcomes like preterm birth, low birth weight and jaundice 14, 15.

While according to WHO, pregnant mothers in developing countries should start ANC during the first three months of pregnancy 7, studies indicate that in many low-income countries early ANC visit is low (24%) compared to 81.9% in the developed countries 16, 17. The prevalence of antenatal visit in Tanzania has gone up from 90% in 2010 to 98% in 2016. Despite this increase, only 24% of the pregnant women are reported to begin ANC visits during the first trimester 18. In many African settings social, family, community context and beliefs are factors identified to affect the health of pregnant women. For example, while some cultures promote special foods and rest for pregnant women, in other societies pregnant women are expected to continue working routinely. In addition, nutritional taboos may deprive pregnant women of essential nutrients particularly iron, protein, vitamin B_12_ and folic acid 19. Mgata and Maluka in their study in Dar es Salaam reported that perceptions of antenatal care, past experience with pregnancy, fear of pregnancy disclosure, socio-cultural beliefs, age, occupation, marital status, parity, behaviour and knowledge level were key individual and social factors for late ANC attendance. In addition, shortage of trained health care workers, lack of spouse's escort and health providers' disrespect of pregnant women were the main health system barriers to early ANC attendance 20. Facility related factors that influence ANC attendance include outcomes for patients, staff satisfaction, equity, efficiency, process quality, humanity, and adherence to external performance targets 21.

The Kigamboni Municipality is among the newly established. There is scarcity of information on ANC attendance (uptake) at the health facilities. Information on the extent of ANC services uptake to determine the extent of late ANC attendance is essential to assist the new Municipality in planning and developing appropriate strategies for early ANC initiation and contribute to the global initiative to reduce maternal and neonatal mortality and morbidity. This study aimed to generate information on the prevalence of late initiation of ANC visits and the associated factors in the new municipality as inputs to scale up plans for timely initiation of ANC visits in order to reduce maternal mortality.

## Methods

### Study site

Kigamboni Municipality is part of Dar es Salam area in Tanzania. The Municipality originated from Temeke Municipal in 2015 under the Government Gazette announcement number 462 of 2015 and effected in 2016. Kigamboni Municipal covers an area of 416 sq. km 22 with an estimated population of 238,591 according to the 2019 population projection. Administratively it has nine wards which are Kibada, Kigamboni, Kimbiji, Kisarawe 2, Mjimwema, Pemba Mnazi, Somangila, Tungi and Vijib weni and 67 sub wards.

The Kigamboni Municipality has both public and private health facilities categorized as hospitals, health centres and dispensaries. The public health facilities include 2 hospitals, 2 health centres and 17 dispensaries; while the private health facilities consist of 1 health centre, 28 dispensaries and 28 clinics. This study involved five public health facilities only from five wards of the municipality including 1 hospital (Vijibweni Hospital), 2 Health centres (Kigamboni and Kimbiji health centres) and 2 Dispensaries (Mji mwema and Kibada dispensaries differentiated according to the national guidelines 23.

### Study design and population

This study employed a cross-sectional analytical study design. The study population included pregnant women in the second and third trimester who attended antenatal care between 2^nd^ September 2021 to 31^st^ October 2021.

### Sample size

The Kish Leslie 24 formula was used to calculate the estimated sample size of 204 based on an estimated 86% proportion of pregnant women with late ANC booking 16 and 0.9% non-response rate and 5% probability margin error.

### Sampling methods

This study employed a stratified sampling method to select the health facilities. Simple random sampling was used to select 1 hospital (Vijibweni Hospital). The two health centres (Kigamboni and Kimbiji) were all included in the study and systematic random approach was used to select the two dispensaries (Mjimwema and Kibada dispensary).

### Data collection tool and procedures

This study used a structured questionnaire to collect data. To ensure quality of the questionnaire and minimize ambiguity, the questionnaire was pre-tested using few participants at the Pemba Mnazi dispensary, not part of the study sites. The questionnaire was prepared in English then translated into Kiswahili. The interviews used the Kiswahili version, a local language commonly spoken in Tanzania.

Data was collected by two research assistants supervised by the researcher. The two research assistants were nurses recruited from the health facilities. All were trained on the purpose of the study, data collection tool and methods, consenting procedures, data management and ethical issues. The questionnaire collected demographic data (age, marital status), clinical information about the current pregnancy (parity and gestation age) and factors associated with late ANC booking (education, occupation and income) were collected by directly asking participants and cross examining them on the information contained in the ANC card.

### Data management and analysis

The data was reviewed on daily basis by the researcher. Data quality including accuracy (well capturing of data), completeness (required information is well documented) and any inconsistence were checked on daily basis. Data entry and analyses were done using Statistical Package for Social Sciences (SPSS) version 25.0. Descriptive statistics were summarised using frequency and percentages, and the associations between the individuals, social-economical and facility related factors with late ANC booking were determined. Multivariate logistic regression was done to determine the Adjusted Odds Ratio of a measure of association between independent and the outcome variables controlling for confounder. The p < 0.05 was considered statistically significance.

### Ethical consideration

The study received ethical clearance from the Hubert Kairuki Memorial University Institutional Review Board (Ref: HKMU/IREC/27.10/154) dated 7^th^ February 2021. Similarly, permission to conduct the study in the selected health facilities was obtained from the Kinondoni Municipality authorities, (Ref: KGMC/2.17/118) dated 26th August 2021. A prepared patient information sheet provided participants with information that the study carried minimum risk and the benefits and voluntariness nature of the study was explained to all participants. Confidentiality was assured by ensuring that participant identifiers were not used to link participants to their names. Participants were recruited into the study once they consented willingly to take part in the study.

## Results

The study recruited 204 pregnant women from the public health facilities in Kigamboni Municipality. Table 1 presents the characteristics of the participants. Majority (64.2%) were in the age group 26-45 years and most of them (72.5%) were married. Less than half (41.2%) had attained secondary education; 39.7% and 38.2% were self-employed and housewives respectively. Less than one third (29.4%) earned less than Tsh 150,000 per month and more than half were on their first or second parity. High proportion (85.3%) of pregnant women had late booking of ANC.

**Table 1:** Characteristic of the pregnant women attending public health facilities of Kigamboni Municipality in Dar Es Salaam Region, Tanzania

Category	N	Percentage (%)
**Age group**		
16 - 25 years	73	35.8
26 - 45 years	131	64.2
**Marital status**		
Married	148	72.5
Divorced	4	2
Separated	11	5.4
Single	38	18.6
Widow	2	1
**Level of education**		
Primary level	83	40.7
Secondary level	84	41.2
Tertiary level	17	8.3
None	19	9.3
**Occupation**		
Employed	29	14.2
Self-employed	81	39.7
Unemployed	14	6.9
Housewife	78	38.2
**Monthly income**		
Less than 150K	60	29.4
Between 150K and 500K	41	20.1
500K	19	9.3
No income	81	39.7
**Parity**		
1-2 births	110	53.9
3-4 births	61	29.9
Prime gravida	33	16.2
**Gestational age at first visit**		
<12 weeks	30	14.7
13+ weeks	174	85.3

### Bivariate analysis results

Table 2 presents the association between factors and late ANC booking. Majority (90.4%) of the respondents with primary school education had late ANC booking compared to 58.8% among those with tertiary education level (p = 0.002). In addition, participants whose parity was above 3 had highest (96.7%) proportion of late ANC booking (p=0.001).

**Table 2:** Bivariate analysis of associated factors for ANC late booking among women attending public health facilities of Kigamboni Municipality in Dar Es Salaam Region, Tanzania

Variable Category	N	% Late booking	p-value
**Age group**			
16-25 years	59	80.8	0.178
26-45 years	115	87.8	
**Marital status**			
Married	148	83.8	
Divorce	4	100	
Separated	11	81.8	0.749
Single	38	89.5	
Widow	2	100	
**Level of education**			
Primary level	83	90.4	
Secondary level	84	85.7	0.002
Tertiary level	17	58.8	
None	19	88.9	
**Employment status**			
Employed	29	72.4	
Self-employed	81	85.2	0.17
Unemployed	14	85.7	
Housewife	78	89.7	
**Monthly income**			
Less than 150K	60	81.7	0.62
Between 150K and 500K	41	87.8	
500K	19	78.9	
No income	81	87.7	
**Parity**			
Primigravida	33	30.9	
1-2 births	110	78.2	0.0001
3-4 births	61	96.7	

### Multivariate analysis results

Table 3 presents the multivariate analysis results of the association of independent variables with late booking. All age groups showed no statistically significant association with late ANC booking. However, participants with tertiary education level had ten times increased likelihood of late ANC booking compared to those with no education [AOR=10.174, (95%CI: 1.002-103); p = 0.050]. Primigravida women when compared to women whose parity is between 1 – 2 had 90% less likelihood of early booking. However, pregnant women with 3 or more parity have significantly increased likelihood of late ANC booking AOR=0.101, 95% (CI: 0.170-0.605) p = 0.012] compared to those with 1 or 2 deliveries. Parity of 1 – 2 did not significantly associated with late ANC booking compared to primigravida [AOR – 0.621, (95%CI: 0.221 – 1.724) p = 0.361)].

**Table 3:** Multivariate analysis association of independent variables with late booking among women attending public health facilities of Kigamboni Municipality in Dar Es Salaam Region, Tanzania

Variable category	Adjusted Odds ratios	95% confidence		Interval	P-value
**Age group**					
16 - 25	1				
26-30	0.345	0.104	1.141		0.079
31-45	0.726	0.164	3.215		0.669
**Marital status**					
Not married	1				
Married	1.933	0.612	6.098		0.261
**Education status**					
No Education	1				
Primary	1.291	0.166	10.032		0.807
Secondary	1.523	0.209	11.203		0.676
Tertiary	10.174	1.002	103.301		0.05
**Employment status**					
Self-employed	1				
Housewife	0.429	0.152	1.216		0.112
Unemployed	0.64	0.101	4.053		0.636
Employed	0.921	0.254	3.327		0.9
**Parity**					
Prime gravida	1				
1 - 2 births	0.621	0.224	1.724		0.361
3 - 4 births	0.101	0.17	0.605		0.012

## Discussion

Late ANC booking is associated with increased morbidity and mortality for both mothers and their new-born. It is common practice that pregnant women should attend ANC from 12th week of pregnancy 25 in line with WHO recommendation 7. We observed in this study a very high proportion (85.3%) of pregnant women who booked late for antenatal care. This proportion is higher than 70.4% reported from Lushoto, Tanzania 18 but similar to previous findings in Dar es Salam 20. Similar high prevalence of late ANC bookings has been reported in the Africa region including Ethiopia 26 - 28, South Africa 11 and also in Malaysia 29. The current high prevalence of late ANC booking in Kigamboni against the general trend of increased ANC attendance in the country is an interesting finding which could be indicating a failure of the existing strategies in motivating pregnant mothers to initiate ANC attendance early. This finding could be overcome by reviewing the existing strategies with a view to determine gaps (e.g., overcrowding at the facilities, inadequate supplies, care-provider attitude and practices), and designing innovative strategies of information and dissemination and institutional and health care workers practice improvement. Also, the results should prompt researchers to carry out studies to determine the availability of the global and national ANC guidelines at the health facilities, and the extent of uptake of the guidelines by health care providers. In addition, the current increased number of health facilities in the country and easy access to routine health care have improved significantly and possibly have reduced the need for pregnant women to frequently seek for such care from ANC. However, overcrowding at health facilities can negatively influence ANC attendance.

In this study, we found that tertiary education and multiparous women had high Odds for late ANC booking. This finding is consistent with previously reported prevalence rate of late ANC booking 30 but differ from reported high Odds of early ANC booking among pregnant women with higher education 31. Education exposes people to information and it is expected that pregnant women with tertiary education are more knowledgeable on the pregnancy and related complications, advantages of ANC services and therefore, would take advantage of ANC services early. Tertiary education, however, is more likely to be associated with employment where busy office schedules would burden pregnant women from finding time to utilise ANC services early, hence the late booking.

We did not find a significant association between employment and late ANC booking in this study. On the contrary, Wolde et al reported that housewives and self-employed mothers had increased odds of late ANC booking than government employed mothers^7^. Their results support previous findings of a study in Tanzania^17^. It was argued that housewives were at increased odds of booking for ANC late because of the workload they do in the house and therefore, lack time to visit health facilities. In this study only 14% of the women were employed compared to about 40% and 38% who were self-employed and housewives respectively. The finding of insignificant association of the influence of employment on ANC booking could be accounted for by the small number of employed participants.

Parity of 1 – 2 in this study did not significantly associate with late ANC booking. However, multigravida (3 – 4) have increased Odds for late ANC initiation. This finding supports previous reports 32 which reported high Odds of late ANC booking among multigravida women. The confidence built among multiparous women from previous pregnancy experiences on pregnancy identification, the period when complications are more likely to occur, and community support systems could be accounting for the late initiation in this group.

## Conclusion

While national statistics indicate that ANC attendance has increased, we found that the proportion of pregnant women who booked late for ANC was high. Contrary to the expectation that high parity and tertiary education would motivate pregnant women to utilise ANC services, these factors in this study significantly associated with late ANC booking. The findings suggest that there could be inadequate motivation strategies for pregnant mothers to early book for ANC, therefore, health education campaigns on the advantages of early ANC booking and the risks associated with late ANC booking should be enhanced. Attempt should be made to review the existing strategies including the guidelines in order to design more innovative approaches that would motivate more pregnant women to book early for ANC.

## Data Availability

All data and materials concerning this research article are available for sharing if needed.
